# West Nile Virus in Farmed Crocodiles, Zambia, 2019

**DOI:** 10.3201/eid2604.190954

**Published:** 2020-04

**Authors:** Edgar Simulundu, Kunda Ndashe, Herman M. Chambaro, David Squarre, Paul Michael Reilly, Simbarashe Chitanga, Katendi Changula, Andrew N. Mukubesa, Joseph Ndebe, John Tembo, Nathan Kapata, Matthew Bates, Yona Sinkala, Bernard M. Hang’ombe, King S. Nalubamba, Masahiro Kajihara, Michihito Sasaki, Yasuko Orba, Ayato Takada, Hirofumi Sawa

**Affiliations:** Author affiliations: The University of Zambia, Lusaka, Zambia (E. Simulundu, S. Chitanga, K. Changula, A.N. Mukubesa, J. Ndebe, B.M. Hang’ombe, K.S. Nalubamba, A. Takada, H. Sawa);; Lusaka Apex Medical University, Lusaka (K. Ndashe);; Hokkaido University, Sapporo, Japan (H.M. Chambaro, D. Squarre, M. Kajihara, M. Sasaki, Y. Orba, A. Takada, H. Sawa);; Ministry of Fisheries and Livestock, Lusaka (H.M. Chambaro, Y. Sinkala);; Department of National Parks and Wildlife, Chilanga, Zambia (D. Squarre);; The University of Edinburgh, Edinburgh, Scotland, UK (D. Squarre);; University of Florida, Florida, USA (P.M. Reilly);; HerpeZ, University Teaching Hospital, Lusaka (J. Tembo, M. Bates);; Zambia National Public Health Institute, Ministry of Health, Lusaka (N. Kapata);; University of Zambia–University College London Medical School, Research and Training Programme, Lusaka (N. Kapata);; University of Lincoln, Lincoln, UK (M. Bates); University College London, London, UK (M. Bates)

**Keywords:** West Nile virus, West Nile fever, Flaviviridae, Crocodylus niloticus, zoonoses, mosquitoborne disease, arboviruses, Zambia, crocodiles, vector-borne infections, viruses

## Abstract

We detected West Nile virus (WNV) nucleic acid in crocodiles (*Crocodylus niloticus*) in Zambia. Phylogenetically, the virus belonged to lineage 1a, which is predominant in the Northern Hemisphere. These data provide evidence that WNV is circulating in crocodiles in Africa and increases the risk for animal and human transmission.

West Nile virus (WNV), the causative agent of West Nile fever (WNF), is an arbovirus of the genus *Flavivirus*, family *Flaviviridae*. WNV has been reported in a variety of species but is maintained mainly between birds and ornithophilic mosquitoes, with incidental transmission to end hosts, such as humans, horses, and other vertebrates (*1*). Recently, WNV was detected in snakes (*2*), and antibodies against WNV were found in farmed crocodiles (*Crocodylus niloticus*) in Israel and Mexico and in alligators (Alligator mississippiensis) in the United States (*3*). Moreover, a severe outbreak of WNV neurologic disease in farmed alligators was reported in Florida (*4*). Meanwhile, the role of reptiles in the epidemiology of WNV remains obscure.

Up to 9 genetic lineages of WNV have been proposed (*5*), but lineages 1 and 2 have been associated with most human outbreaks of neurologic disease. Lineage 1 is globally distributed, and major outbreaks involving this lineage have been reported in the Americas, Europe, Asia, Oceania, and North Africa (*5*). In contrast, lineage 2 was reported exclusively in southern Africa and Madagascar until the 2000s, when it emerged in Europe (*6*). Although lineage 2 is predominant in southern Africa, WNV lineage 1 was detected in a pregnant mare in South Africa in 2010 (*7*).

In March 2019, a crocodile farm in Southern Province, Zambia, reported that some yearlings had exhibited clinical signs including anorexia, weakness, swimming in circles, bloody diarrhea, and scoliosis and were euthanized. Postmortem examination revealed congestion of the lungs, hemorrhagic intestines and trachea, and hydropericardium. Clinical signs and postmortem findings led to the suspicion of WNF, coccidiosis, salmonellosis, or enterotoxemia. We collected 11 whole blood samples from the postoccipital sinus of the spinal vein of affected crocodiles for molecular detection of WNV. 

We used a QIAamp Viral RNA Mini Kit (QIAGEN, https://www.qiagen.com) to extract total RNA and the OneStep RT-PCR Kit (QIAGEN) to detect part of the WNV genome by using the primer pair WNNY-514 (5′-CGG CGC CTT CAT ACA CW-3′) and WNNY-905 (5′-GCC TTT GAA CAG ACG CCA TA-3′). These primers amplified an ≈400 bp fragment in 2/11 samples tested, which we then used for direct Sanger sequencing. The 2 nucleotide sequences we obtained were 100% identical to each other, and a BLAST analysis (https://blast.ncbi.nlm.nih.gov) showed 99% sequence identity to WNV isolate ArD76986/1990/SN (GenBank accession no. KY703854), which was detected from *Culex poicilipes* mosquitoes in Senegal (*5*). To obtain the complete sequence of the polyprotein gene, we designed several overlapping primers (Appendix Table) to use in reverse transcription PCR (RT-PCR) assays and sequencing. We deposited the sequence, Croc110/2019/ZM, in GenBank (accession no. LC489409).

For phylogenetic analysis, we aligned complete polyprotein amino acid sequence of Croc110/2019/ZM and reference sequences from GenBank by using MUSCLE (http://www.drive5.com/muscle). We constructed the phylogenetic tree in MEGA6 (https://www.megasoftware.net) by using the maximum-likelihood method and the Jones-Taylor-Thornton matrix-based model with 1,000 bootstrap replicates. Phylogenetic analysis revealed that Croc110/2019/ZM belonged to lineage 1a (Figure) and was most closely related to a WNV isolate from a camel in the United Arab Emirates (GenBank accession no. KU588135) and isolate ArD76986/1990/SN from Senegal (accession no. KY703854).

**Figure:**
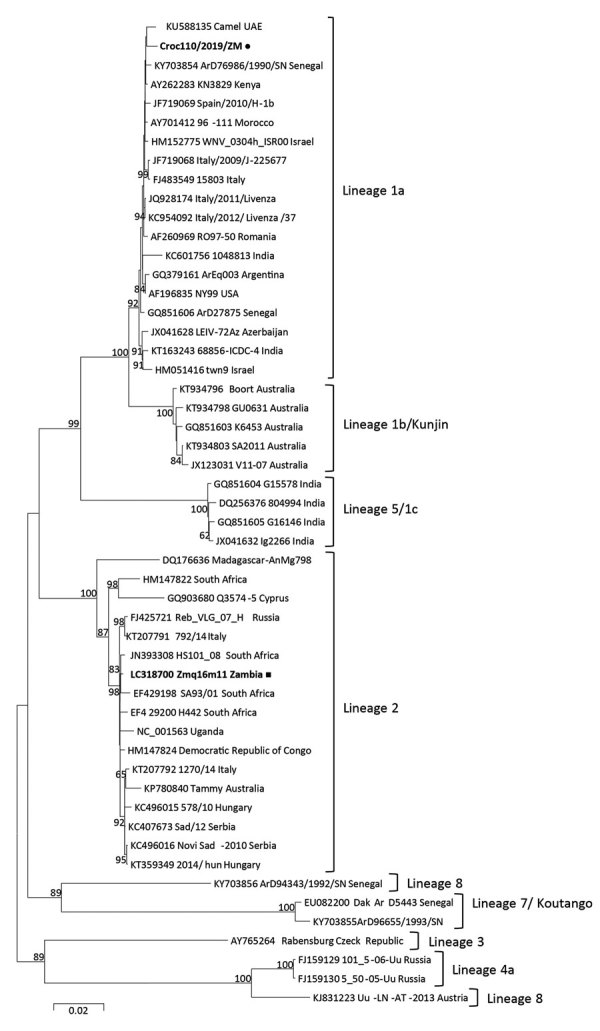
Phylogenetic tree of complete polyprotein amino acid sequences of West Nile virus (WNV) from farmed crocodiles, Zambia (black dot), and reference sequences. Phylogenetic analysis was conducted by using the maximum likelihood method based on the JTT matrix-based model with 1,000 bootstrap replicates using MEGA6 software (https://www.megasoftware.net). Bootstrap values >60% are shown next to the branches. The analysis involved 52 amino acid sequences; there were a total of 3,415 positions in the final dataset. All positions containing gaps and missing data were eliminated. Reference sequences included in the analysis are shown with their GenBank accession numbers, strain name or source, and country of origin. Black square indicates WNV previously detected from mosquito in the Western Province of Zambia. Scale bar indicates nucleotide substitutions per site.

Our study confirmed WNV infection in farmed crocodiles in Africa. Clinical signs and pathological changes in multiple organs correlated with those described for WNV infection in farmed American alligators in the United States (*4*). The source of the WNV in the outbreak remains unresolved. WNV might have been circulating on the farm because the farmer indicated that lymphohistiocytic proliferative cutaneous lesions were observed in the crocodiles for some time. Such lesions can be associated with WNV infection and cause considerable economic losses because of lowered skin quality (*8*). Transmission among the crocodiles could occur orally from cannibalism and by cloacal shedding of WNV from infected animals (*8*).

Phylogenetic analysis of the complete polyprotein amino acid sequence of Croc110/2019/ZM grouped the virus in lineage 1a. Previous mosquito surveillance studies in Western Province, Zambia, identified WNV lineage 2 from *Culex quinquefasciatus* mosquitoes (*9*). Our findings suggest that multiple WNV lineages are co-circulating in Zambia and that multiple host species could be involved.

WNF outbreaks have not been reported in humans in Zambia, but 10.3% of 3,625 persons who participated in a serosurvey were seropositive for WNV in Western and North-Western provinces of the country (*10*), suggesting possible WNV infection. Detection of WNV in mosquitoes in Western Province and our finding of the virus in crocodiles in Southern Province suggest that WNV could be a neglected emerging infectious pathogen and might be associated with WNF in animals and humans in Zambia. Our study stresses the need for increased clinical awareness among veterinary and medical practitioners and continued monitoring of WNV in vectors and animals, including reptiles, to clarify the ecology and life cycle of this pathogen, particularly in regions where WNF is poorly understood.

Appendix Additional information on West Nile virus in crocodiles, Zambia.

## References

[1] Hayes EB, Komar N, Nasci RS, Montgomery SP, O’Leary DR, Campbell GL. Epidemiology and transmission dynamics of West Nile virus disease. Emerg Infect Dis. 2005;11:1167–73. 10.3201/eid1108.050289a 16102302 PMC3320478

[2] Dahlin CR, Hughes DF, Meshaka WE Jr, Coleman C, Henning JD. Wild snakes harbor West Nile virus. One Health. 2016;2:136–8. 10.1016/j.onehlt.2016.09.003 28616487 PMC5441359

[3] Ariel E. Viruses in reptiles. Vet Res (Faisalabad). 2011;42:100. 10.1186/1297-9716-42-100 21933449 PMC3188478

[4] Jacobson ER, Ginn PE, Troutman JM, Farina L, Stark L, Klenk K, et al. West Nile virus infection in farmed American alligators (*Alligator mississippiensis*) in Florida. J Wildl Dis. 2005;41:96–106. 10.7589/0090-3558-41.1.96 15827215

[5] Fall G, Di Paola N, Faye M, Dia M, Freire CCM, Loucoubar C, et al. Biological and phylogenetic characteristics of West African lineages of West Nile virus. PLoS Negl Trop Dis. 2017;11:e0006078. 10.1371/journal.pntd.0006078 29117195 PMC5695850

[6] Bakonyi T, Ivanics E, Erdélyi K, Ursu K, Ferenczi E, Weissenböck H, et al. Lineage 1 and 2 strains of encephalitic West Nile virus, central Europe. Emerg Infect Dis. 2006;12:618–23. 10.3201/eid1204.051379 16704810 PMC3294705

[7] Venter M, Human S, van Niekerk S, Williams J, van Eeden C, Freeman F. Fatal neurologic disease and abortion in mare infected with lineage 1 West Nile virus, South Africa. Emerg Infect Dis. 2011;17:1534–6. 10.3201/eid1708.101794 21801644 PMC3381566

[8] Lott MJ, Moore RL, Milic NL, Power M, Shilton CM, Isberg SR. Dermatological conditions of farmed Crocodilians: A review of pathogenic agents and their proposed impact on skin quality. Vet Microbiol. 2018;225:89–100. 10.1016/j.vetmic.2018.09.022 30322539

[9] Orba Y, Hang’ombe BM, Mweene AS, Wada Y, Anindita PD, Phongphaew W, et al. First isolation of West Nile virus in Zambia from mosquitoes. Transbound Emerg Dis. 2018;65:933–8. 10.1111/tbed.12888 29722174

[10] Mweene-Ndumba I, Siziya S, Monze M, Mazaba ML, Masaninga F, Songolo P, et al. Seroprevalence of West Nile Virus specific IgG and IgM antibodies in North-Western and Western provinces of Zambia. Afr Health Sci. 2015;15:803–9. 10.4314/ahs.v15i3.14 26957968 PMC4765448

