# Supplementary material for: West Nile Virus in Farmed Crocodiles, Zambia, 2019
Source: Emerg Infect Dis. 2020 Apr;26(4):811–4. doi: 10.3201/eid2604.190954 (PMC7101096; doi:10.3201/eid2604.190954)
Supplement: Appendix — Additional information on West Nile virus in crocodiles, Zambia. [file 19-0954-Techapp-s1.pdf]

Article DOI: https://doi.org/10.3201/eid2604.190954

West Nile Virus in Farmed Crocodiles, Zambia, 2019

Appendix

Appendix Table. List of primers used for reverse transcription-PCR and sequencing of full-length West Nile virus polyprotein gene in farmed crocodiles, Zambia

Serial no.	Primer name	Primer sequence
1	WNV_1F	AGTAGTTCGCCTGTGTGAGC
2	WNV_425R	ACGTCAGTAGCATTTACCGTCA
3	WNV_1020R	TGCTCCAGATACTCCCTCCA
4	WNV_806F	GCACCAAGGCCACAAGGTAT
5	WNV_2001R	TGAAGCCACTGACGAGATGG
6	WNV_2291R	TCAATGCTCGTGACAGGTCC
7	WNV_1810F	ACGTCGGGTCATTTGAAGTGT
8	WNV_2876R	AACATCCGAGTGCTGGTGAG
9	WNV_2976R	CAGGAACATCCGAGTGCTGG
10	WNV_2760F	CAAGTCGGCACCTAAACGTC
11	WNV_3273F	AACATCCGAGTGCTGGTGAG
12	WNV_3766R	AAACAGCCGCCAACATCAAC
13	WNV_3949R	TCATCCAAGCTACTGCCAGTG
14	WNV_3719F	TCGCAGAATCTAATTCGGGAGG
15	WNV_4903R	TCATCTGCACCTCATCCTGC
16	WNV_4665F	GACCACCGGAGTCTACAGGA
17	WNV_5870R	GCCTTGAAGTTAGCCCCCAT
18	WNV_5640F	GGCCTGGAACTCTGGATACG
19	WNV_6806R	AGAGAAGGGAAAGCAGCAACA
20	WNV_6616F	GCTCTCCAGACAATTGCCTT
21	WNV_7147R	CGGTGAGGGTGACTTGTCC
22	WNV_7815R	TTTCCTGGCGTGTTTTGCTG
23	WNV_7639F	TGGACACTCATAAAGAACATGG
24	WNV_8841R	CAGAAACGCCCACAACCAAT
25	WNV_8641F	TCGCTGGTTAATGGAGTGGT
26	WNV_9402F	CAAGTTGTCACCTACGCCCT
27	WNV_9816R	TGGAACCTGCTGCCAATCAT
28	WNV_9649F	GAAGAGAGACTCAGCCGCAT
29	WNV_9850F	ACCGCTTGTCTGGCTAAGTC
30	WNV_10842R	GCCGTGCACTTCAAACCG
31	WNV_10267F	ACGTGGGCAGAAAACATCCA
31	WNV_10295R	ACTGTGTCTTCAACCAAAGT
33	WNV_11012R	ACCACCAGCCACCATTGTC
